# Integrated neuromuscular training intervention applied in schools induces a higher increase in salivary high molecular weight adiponectin and a more favorable body mass index, cardiorespiratory fitness and muscle strength in children as compared to the traditional physical education classes

**DOI:** 10.3389/fpubh.2024.1337958

**Published:** 2024-05-02

**Authors:** Fidanka Vasileva, Raquel Font-Lladó, Gemma Carreras-Badosa, Jorge Cazorla-González, Abel López-Bermejo, Anna Prats-Puig

**Affiliations:** ^1^Pediatric Endocrinology Research Group, Girona Institute for Biomedical Research, Girona, Spain; ^2^University School of Health and Sport, University of Girona, Girona, Spain; ^3^Research Group of Culture and Education, Institute of Educational Research, University of Girona, Girona, Spain; ^4^Department of Medical Sciences, University of Girona, Girona, Spain; ^5^Pediatric Endocrinology, Dr. Josep Trueta Hospital, Girona, Spain; ^6^Research Group of Clinical Anatomy, Embryology and Neuroscience, Department of Medical Sciences, University of Girona, Girona, Spain

**Keywords:** integrated neuromuscular training, schools, high molecular weight adiponectin, saliva, children, cardio-metabolic health

## Abstract

**Background:**

High-molecular-weight adiponectin (HMW-adiponectin) is a cardio-metabolic health protector. Objectives: (1) to compare body mass index (BMI), cardiorespiratory fitness (CRF) and muscle strength (MS) in healthy school-children depending on their baseline salivary-HMW-adiponectin concentration; and (2) to apply a 3-month integrated neuromuscular training (INT) and evaluate its effects on salivary-HMW-adiponectin concentration, BMI, CRF and MS in the same children. Additional goal: to identify if any potential changes during the 3-month period may be related to a potential change in salivary-HMW-adiponectin concentration.

**Methods:**

Ninety children (7.4 ± 0.3 years) were recruited in primary schools and randomly allocated into control or intervention group. The intervention consisted of a 3-month INT applied during physical education (PE) classes, twice-weekly, while the control group had traditional PE classes. Body mass and height were measured, BMI was calculated and HMW-adiponectin was quantified in saliva. To assess CRF and MS, 800 m-run and hand-dynamometry were applied, respectively. All measurements were performed twice, at baseline and after 3 months.

**Results:**

Children with higher baseline salivary-HMW-adiponectin have more favorable BMI (*p* = 0.006) and slightly higher CRF (*p* = 0.017) in comparison to the children with lower baseline salivary-HMW-adiponectin. There were no big changes after the 3-month-period neither in the control, nor the INT group. However, it is worthy to note that the INT induced slightly higher increase in salivary-HMW-adiponectin (*p* = 0.007), and a slightly higher improvement in BMI (*p* = 0.028), CRF (*p* = 0.043) and MS (*p* = 0.003), as compared to the traditional PE classes. Finally, the INT-induced improvement in CRF was associated with the increased post-salivary-HMW-adiponectin concentration (*p* = 0.022).

**Conclusion:**

Main findings may suggest the potential utility of an INT as a cost-effective strategy that can be applied in schools to induce cardio-protective effects in school-children.

## 1 Introduction

Adiponectin is a protein encoded by the *Adipoq* gene in humans, mainly produced by the adipose tissue (1, 2), but it can also be secreted by the skeletal muscle, cardiac muscle, salivary gland epithelial cells and other tissues (2–4). Salivary adiponectin is involved in the regulation of the local immune response (5). Nowadays, salivary adiponectin is becoming a promising diagnostic marker for cardio-metabolic diseases especially in children, mainly because saliva collection is a non-invasive diagnostic procedure (6), and it does not require specially trained personnel for sampling procedure (7). Even though there is a modest degree of overlap between salivary and plasma proteomes (only 27%), the analysis of distribution across Gene Ontology categories, including molecular function, biological processes and cellular components, revealed some similarities (8). Moreover, it has been shown that approximately 40% of the proteins identified as candidate markers for cardiovascular diseases, stroke and cancer were detectable in saliva (8). In addition, previous studies reported a weak correlation between salivary and plasma adiponectin levels in patients with metabolic syndrome, and a moderate correlation in healthy adult population, thus requesting further attention in this topic and exploration of the potential utility of salivary adiponectin in diagnostic purposes (9, 10).

Adiponectin can be found in different molecular weight forms among which high-molecular-weight (HMW) adiponectin is considered to be the most biologically active form in terms of glucose homeostasis (11). Moreover, HMW-adiponectin has been shown to be a cardio-metabolic health protector due to its vasodilator, anti-apoptotic and anti-inflammatory effects (12, 13). It acts as a messenger providing an inter-organs crosstalk, regulating lipid and glucose metabolism, modulating thermogenesis and energy expenditure, and increasing insulin sensitivity (1, 14). High HMW-adiponectin levels are related to more favorable body composition (15), whereas overweight individuals, individuals with obesity, and cardiovascular disease patients have low HMW-adiponectin levels (16, 17).

Interestingly, an increase in HMW-adiponectin concentration was observed after acute and chronic physical exercise with intensities ranging from moderate to vigorous (12, 18–20). In addition, higher circulating adiponectin was reported in physically active compared to inactive individuals (21). Therefore, HMW-adiponectin is considered as a potential marker mediating the health-beneficial effects induced by physical exercise (14). However, recent studies provided inconsistent evidence, reporting unchanged adiponectin levels after exercise (14, 22).

Integrated neuromuscular training (INT) is a type of physical exercise—a specifically tailored training program with moderate intensity that improves health and fundamental motor skills (23–25). It has also been recognized as an innovative approach for school-aged children, and an effective strategy for enrichment of their motor learning experience through the combination of efficient cognitive processing, correct movement patterns, and muscle force production (24). The combination of these components may result in improved physical fitness (24). One component of physical fitness is cardiorespiratory fitness (CRF), which is mainly determined by the capacity of the cardio-respiratory system to deliver oxygen to the working muscles while exercising (26). Another component is muscle strength which is defined as the capacity of a muscle, or group of muscles, to exert force under a given set of conditions (27).

Based on the previous findings, we postulated the following hypothesis and objectives for the present study:

1. Because previous evidence has related plasma HMW-adiponectin with more favorable body composition in adults (15), we hypothesize that school-aged children with higher baseline salivary HMW-adiponectin may also present more favorable body mass index (BMI) in comparison to the children with lower baseline salivary HMW-adiponectin;
2. Because recent studies reported unchanged adiponectin levels after physical exercise (14, 22) but others reported increased HMW-adiponectin after acute and chronic physical exercise with intensities ranging from moderate to vigorous (12, 18–20), we hypothesize that a 3-month moderate intensity INT applied during physical education (PE) classes may potentially induce a more pronounced increase in salivary HMW-adiponectin in healthy school-aged children than the traditional PE classes.
3. Because an 8-week INT significantly improved physical fitness in children (24), we hypothesize that a 3-month INT intervention applied during PE classes will also induce higher improvement in CRF and muscle strength in comparison to the traditional PE classes.

Therefore, our objectives were: (1) to compare BMI, CRF and muscle strength in healthy school-aged children depending on their baseline salivary HMW-adiponectin concentration; and then (2) to apply a 3-month INT intervention in schools and evaluate its effects on salivary HMW-adiponectin concentration, BMI, CRF and muscle strength in the same children. Additional goal was to identify if any potential changes in terms of BMI, CRF and muscle strength may be related to a potential change in salivary HMW-adiponectin concentration after the 3-month period.

## 2 Methods

### 2.1 Population and ethics

A total of 90 apparently healthy children (44 boys and 46 girls; 7.4 ± 0.3 years) were recruited in schools in Cassà de la Selva and Salt (Girona, Northeastern Spain). Schools were randomly allocated into control (*N* = 45) or INT (*N* = 45) group. Note that children randomization within the same school and during the same PE class was not possible due to ethical reasons, thus we randomly allocated schools as a control on INT group. However, following the completion of the study, the INT was offered to the control school as well. This decision was made to ensure that all children that participated in the study were treated fairly and equally, prevent any perception of discrimination that may have been provoked by the schools randomization process, and finally comply with the ethical guidelines and the good practices in research. Inclusion criteria were: (1) no evidence of chronic or acute illness in the month preceding potential enrollment; and (2) age between 7 and 9 years. Exclusion criteria were: (1) major congenital abnormalities; (2) illness or chronic use of medication; (3) musculoskeletal, neurological disorder and/or certain medication therapy that could alter postural stability and cardiorespiratory function; and finally (4) attending fewer than 80% of the PE classes. The research was approved by the Institutional Review Board of Dr. Josep Trueta Hospital, Girona, Spain (CEIm:2016.134). Signed consent was obtained from the parents of all children included in the study. All measurement procedures and sample collection were conducted on the same day following enrollment and after obtaining signed consent from parents of all participating children. Prior to any measurements, the children underwent familiarization with the test protocols. Only 4% of the children participating in the study did not comply with the recommendations for physical activity at baseline (≥1 h/day) (28). Even though most of the children complied with physical activity recommendations representing a homogenous sample, we observed some heterogeneity in terms of type of sport practiced as extracurricular activity (e.g., biking, basketball, swimming etc.).

Worthy to note is that both schools that participated in this study were in the same province and country, thus ensuring the homogeneity of the curricular content for the PE classes. Prior to the start of the study, the PE teacher that would deliver the INT together with the researcher-expert in INT (25) was familiarized with the intervention and trained to deliver the INT sessions.

### 2.2 Intervention

PE classes in both groups (control and INT) were held twice per week with duration of 60 min and the following structure: introductory segment (20 min), main segment (30–35 min), and a concluding segment (5–10 min). The main and the concluding segments of the PE classes were the same in both groups. The difference between the control and the INT group was only in the introductory segment of the PE classes, where the INT group received the INT as a warm-up activity, while the control group did not.

During the introductory segment of the PE classes, the control group followed the traditional warm-up consisted of activities designed to prepare the cardiovascular system for the up-coming effort during the class and exercises that increased the range of motion of the particular joints and parts of the body which would be predominantly engaged during the main segment of the PE class (29). On the other hand, the INT group received the INT as a warm-up activity for 3 months. The INT consisted of 24 sessions applying strength, coordination, dynamic stabilization, plyometrics, speed and agility exercises, organized in a progressive circuits and gamified sessions with moderate intensity (25). All sessions were delivered by the previously trained PE teacher and the researcher-expert in INT (25). After the 20-min warm-up during the introductory segment of the class, children continued with the main segment of the PE class.

The main segment of the PE classes in both groups consisted of didactic delivery of the specific curricular content which is outlined in the national curricula for PE, by the PE teacher: (1) aerobic activities (running, jumping a rope) and activities that involve solving motor tasks in environmental conditions (outdoors circuits and polygons, orienteering activities); (2) activities that will induce development of fundamental motor skills, motor abilities and motor competence (motor challenges that contain elements from individual sports: athletics, gymnastics, tennis); (3) activities that will induce development of interaction skills and team-work (cooperative motor challenges that contain elements from sport games: football, basketball, handball, volleyball); (4) traditional and contemporary dances; and (5) outdoor activities in the natural environment (hiking, cycling, rollerblading, skating).

Finally, the concluding segment of the PE classes in both groups consisted of a short period with less dynamic activities that allowed children to recuperate and gradually bring their heart rate back to normal. In the concluding segment, the children had an opportunity to address any potential doubts that surged during the class with their teacher, to reflect on the content covered and the newly acquired skills, as well as to prepare for the following class.

### 2.3 Biological samples collection

Saliva samples were collected in the morning between 8.00 and 10.00 a.m. in a fasting state and stored at −20°C following the manufacturer’s protocol. Participants had to discharge 1–4 mL of saliva into the 5 mL polystyrene specimen tube with lid, after natural accumulation in the oral cavity. Note that children were not allowed to drink water and brush their teeth before sampling (per manufacturer’s protocol) with the aim to prevent potential cofounding effects that may be induced by stimulated saliva sampling. The same procedure was performed twice, at baseline and after 3 months.

### 2.4 Anthropometric measurements

Anthropometric measurements were performed in the schools at the morning hours (between 8.00 and 10.00 a.m.). Body mass was measured through bioelectric impedance analysis using a calibrated digital scale (Portable TANITA, 240MA, Amsterdam, Netherlands). Participants were instructed to stand barefoot on the Tanita 240MA platform wearing light clothes. The researcher in charge was responsible to ensure proper positioning of the participants during the measurement. Following initiation of the measurement, participants remained still until the measurement was complete and their body mass was recorded in kg with the accuracy of one decimal place. Height was measured with a wall-mounted stadiometer (SECA SE206, Hamburg, Germany). Participants were instructed to stand barefoot with their heels against the backboard. The researcher in charge ensured that participant’s head was positioned in the Frankfurt plane, and recorded the height in cm. BMI was calculated as body mass in kg divided by the square of height in m. Age-and sex-adjusted standard deviation scores (SDS) for body mass, height and BMI were calculated using regional normative data (30). All measurements were performed twice, at baseline and after 3 months.

### 2.5 CRF

CRF was assessed by means of a 800 m run test at the end of the measurement sessions (31). The goal was to complete the 800 m running course in a quickest possible time. All participants performed the test at the same time. Before initiation of the test, participants were instructed to maintain a steady pace and run the distance as fast as possible. The test started after a signal (sound of a whistle). Measurement of timing began together with the signal, i.e., when participants start running and ended when they crossed the finish line. The 800 m running course was monitored by 8 researchers with previous experience in CRF evaluation. Participants were being motivated to do their best during the running course. All participants successfully finished the course and the total time to run the course was recorded manually with a stopwatch (in min) by the researchers with previous experience in CRF evaluation. The CRF evaluation was performed twice, at baseline and after 3 months.

### 2.6 Muscle strength

Muscle strength was assessed by means of an analog hand dynamometer after the anthropometric measurements and saliva collection (TKK 5001, Grip-A, Takei, Tokyo, Japan). The dynamometer grip span was adjusted to 5 cm to accommodate children’s hand size, allowing participants to comfortably grasp the dynamometer with their fingers wrapped around the handle and the base placed on the heel of their palm. Children were instructed to hold the dynamometer with their upper arm and forearm forming an angle of approximately 90° while keeping their elbow beside their body, and to squeeze with maximum isometric force for 5 s (32). The researcher monitored the test to ensure proper execution and positioning of the dynamometer, as well as encouraged children to exert maximum effort. The test was repeated if any adjustments or unnecessary movements were observed and the result was recorded (in kg). The muscle strength evaluation was performed twice, at baseline and after 3 months.

### 2.7 Protein concentration quantification

Before initiation of the study, we used a GRANMO 7.12 program to identify the sample size for inclusion based on a previous study including protein quantification. Accepting an alpha risk of 0.05 and a beta risk of 0.2 in a two-sided test, the estimated sample size for the present study was 30 subjects in each group (33).

HMW-adiponectin was assessed in saliva using the Human HMW-adiponectin ELISA kit (CSB-E13400h; Gentaur, Belgium). Sandwich enzyme-linked immunosorbent assay method was applied with an anti-HMW-adiponectin polyclonal antibody as detection antibody. First, the assay components, samples and standards were prepared according to the manufacturer’s instructions. Then, they were loaded onto the pre-coated microplate wells and incubated according to the kit protocol. After the appropriate incubation periods, the wells were washed and the detection reagent was added, accompanied by the substrate solution. Finally, the reaction was stopped as indicated in the protocol, the absorbance was measured at the specified wavelength using a microplate reader, and HMW-adiponectin concentration was calculated following manufacturer’s instructions. Lower detection limit was 0.5 ng/mL and intra-and interassay CVs <4%.

### 2.8 Statistical analysis

Data were analyzed with the statistical package SPSS version 22.0 (SPSS Inc., Chicago, IL, United States). The normality of the data distribution was tested by the Kolmogorov–Smirnov test. Non-normally distributed variables were logarithmically transformed to improve the distribution symmetry. Tertiles were created to compare BMI, CRF and muscle strength among children depending on their baseline salivary HMW-adiponectin concentration. Then, Kruskal Wallis test with *post hoc* pairwise comparisons was applied to examine the differences between children with low, medium and high baseline salivary HMW-adiponectin concentration. Before starting the intervention and after schools were randomly allocated to control or INT group, a t-test, Mann–Whitney U or Chi-squared tests were performed to assure that the two groups of children were comparable at baseline. To assess if an INT induced changes in salivary HMW-adiponectin, BMI, CRF and muscle strength, we first calculated the percentage change of the assessed components in the control and the INT group. The percentage change was calculated as pre-value (baseline) subtracted by the post-value (after 3 months), divided by the pre-value (baseline), and then multiplied by 100. Then, a t-test was applied to test the difference in percentage change induced by the traditional PE classes and the INT. Finally, to identify if changes in terms of BMI, CRF and muscle strength were associated with the post-salivary HMW-adiponectin concentration after the 3-month period, we applied multiple linear regression analyses adjusting for potential confounding variables such as age, sex, BMI and baseline salivary HMW-adiponectin.

## 3 Results

Descriptive characteristics of the studied population according to tertiles of baseline salivary HMW-adiponectin, as well as comparison between children with low, medium and high salivary HMW-adiponectin are presented in Table 1. From the results presented, it can be observed that children with higher baseline salivary HMW-adiponectin have lower body mass SDS (*p* = 0.032), lower BMI SDS (*p* = 0.007) and slightly higher CRF (*p* = 0.017), in comparison to children with lower baseline salivary HMW-adiponectin. No significant differences were observed in height and muscle strength between groups.

**Table 1:** Baseline characteristics according to tertiles of HMW-adiponectin concentration and comparison between the groups of low, medium and high salivary HMW-adiponectin concentration.

	*N* = 90	Low HMW adiponectin (*N* = 30)	Medium HMW adiponectin (*N* = 30)	High HMW adiponectin (*N* = 30)	*p*-value
HMW-adiponectin (ng/mL)	5.95 (3.14–9.04)	2.06 (1.20–3.45)†	6.45 (5.43–7.82)†	11.20 (9.26–14.58)†	**<0.0001**
Age (years)	7.4 ± 0.3	7.4 ± 0.3	7.5 ± 0.3	7.5 ± 0.4	0.922
Sex (m/f)	44/46	13/17	12/18	19/11	0.322
Body mass (kg)	25.7 ± 4.2	26.3 ± 5.1	26.1 ± 2.8†	24.7 ± 4.1†	**0.040**
Body mass SDS	−0.28 (−0.80–0.13)	−0.10 (−0.66–0.34)	−0.21 (−0.71–0.13)†	−0.45 (−1.19–0.01)†	**0.032**
Height (cm)	126 ± 6	126 ± 6	128 ± 7	126 ± 5	0.466
Height SDS	−0.10 (−0.56–0.87)	0.13 (−0.50–0.90)	0.19 (−0.56–0.96)	−0.33 (−0.57–0.60)	0.533
BMI (kg/m^2^)	16.2 ± 1.9	16.6 ± 2.4	16.5 ± 1.2†	15.5 ± 1.8†	**0.006**
BMI SDS	−0.40 (−0.74–0.02)	−0.40 (−0.76–0.20)	−0.23 (−0.58–0.05)†	−0.56 (−0.98–0.30)†	**0.007**
CRF (min)*	5.31 ± 0.57	5.44 ± 0.70†	5.23 ± 0.54†	5.21 ± 0.26†	**0.017**
Muscle strength (kg)	10.08 ± 1.97	9.57 ± 2.17	9.94 ± 1.77	10.39 ± 1.59	0.120

Data for Gaussian variables is presented as mean ± standard deviation. Data for non-Gaussian variables is presented as median and interquartile range. *p*-value is from Kruskal-Wallis test. The *p*-value for categorical variables is from Chi-squared test. Significance level is set at 0.05 and significant values are marked in bold. Significant differences in post hoc test for pairwise comparisons are presented with †. BMI, body mass index; CRF, cardiorespiratory fitness; HMW, high molecular weight; SDS, standard deviation score; * variable with an opposite metric orientation.

Descriptive characteristics of the children and comparison between the control and the INT group at baseline are presented in Supplementary Table S1. Based on the results in Supplementary Table S1, we may conclude that the control and the INT group were comparable at baseline because there are no statistically significant differences between them in any of the studied components (*p* > 0.05; Supplementary Table S1).

Participants’ characteristics at baseline, after 3 months and comparison of the percentage change between the control and the INT group are presented in Table 2. In general, we did not observe big changes after the 3-month period neither in the control, nor in the INT group (Table 2). However, it appears that the changes observed after the 3 months were slightly higher in the INT group as compared to the control group (Table 2). For instance, higher increase in salivary HMW-adiponectin concentration was observed in the children who performed the INT during PE classes, compared to the children who did not (15.47% more, *p* = 0.007). It seems that the INT also induced a higher decrease in body mass (−1.51% more, *p* = 0.004) and BMI (−0.48% more, *p* = 0.028) in comparison to the traditional PE classes. However, these results must be interpreted with caution because the small changes in body mass and BMI in both groups may also be affected by growth. Finally, a higher improvement in CRF (−0.15% more, *p* = 0.043) and muscle strength (9.04% more, *p* = 0.003) were observed after the INT as compared to the traditional PE classes. No significant differences between changes in the control and the INT group were observed in terms of height of the children (Table 2).

**Table 2:** Participants’ characteristics at baseline, after 3 months and comparison of the Δ % between the control and the INT group.

	Control group (*N* = 45)			INT group (*N* = 45)			
	Baseline	After 3 months	Δ %	Baseline	After 3 months	Δ %	*p*-value
HMW-adiponectin (ng/mL)	7.17 (3.56–9.42)	7.26 (2.68–10.97)	21.56	5.66 (2.34–8.32)	7.88 (3.64–10.20)	37.03	**0.007**
Body mass (kg)	25.60 ± 3.81	25.51 ± 3.10	−1.79	25.72 ± 4.70	25.12 ± 3.45	−3.30	**0.004**
Height (cm)	126.37 ± 6.41	127.86 ± 6.34	0.99	126.36 ± 5.84	127.61 ± 6.37	1.30	0.067
BMI (kg/m^2^)	16.14 ± 1.68	15.98 ± 1.46	−0.19	16.23 ± 2.28	15.94 ± 1.62	−0.67	**0.028**
CRF* (min)	5.33 ± 0.49	5.30 ± 0.50	−0.20	5.29 ± 0.62	5.20 ± 0.76	−0.35	**0.043**
Muscle strength (kg)	10.46 ± 2.25	11.32 ± 2.27	1.52	9.76 ± 2.19	11.99 ± 1.97	10.56	**0.003**

*p*-value is from *t*-test comparing Δ % between control and INT group. Significance level is set at 0.05 and significant values are marked in bold. BMI, body mass index; CRF, cardiorespiratory fitness; HMW, high molecular weight; INT, integrated neuromuscular training; Δ %, percentage change (post-value versus pre-value); * variable with an opposite metric orientation.

Beta values, *p*-values and adjusted R^2^ of multiple linear regression analyses representing the associations between the changes in BMI, CRF and muscle strength after the 3-month period with the post-salivary HMW-adiponectin concentration are presented in Table 3. According to the results in Table 3, it seems that none of the changes in the control group was associated with the post-salivary HMW-adiponectin concentration (Beta = −0.394 to Beta = 0.003, *p* = 0.993 to *p* = 0.061, adjusted R^2^ = 0.120 to adjusted R^2^ = 0.779). However, in the INT group the slight improvement in CRF was positively associated with the increased post-salivary HMW-adiponectin concentration (Beta = −0.335, *p* = 0.022, adjusted R^2^ = 0.336). Furthermore, the improvement in CRF induced by the INT explained 34% of the variance of post-salivary HMW-adiponectin concentration. Finally, there were no associations between the change in BMI and muscle strength with post-salivary HMW-adiponectin concentration (Beta = 0.095, *p* = 0.527, adjusted R^2^ = 0.251; Beta = −0.028, *p* = 0.855, adjusted R^2^ = 0.232 respectively; Table 3).

**Table 3:** Regression analyses representing the associations of the Δ % in BMI, CRF and muscle strength with post-salivary HMW-adiponenctin concentration.

	HMW-adiponectin post log					
	Control group (*N* = 45)			INT group (*N* = 45)		
	Beta	*p*-value	Adjusted R squared	Beta	*p*-value	Adjusted R squared
BMI Δ %	−0.394	0.061	0.120	0.095	0.527	0.251
CRF* Δ %	0.003	0.993	0.501	−0.335	**0.022**	0.336
Muscle strength Δ %	−0.371	0.544	0.779	−0.028	0.855	0.232

Adjusted for age, sex, BMI and baseline salivary HMW-adiponectin. Significance level is set at 0.05 and significant values are marked in bold. BMI, body mass index; CRF, cardiorespiratory fitness; HMW, high molecular weight; INT, integrated neuromuscular training; Δ %: percentage change (post-value versus pre-value); *: variable with an opposite metric orientation.

## 4 Discussion

Main findings of the present study are indicating that children with higher baseline salivary HMW-adiponectin have more favorable BMI and slightly higher CRF than children with lower baseline salivary HMW-adiponectin. Furthermore, we observed only modest changes during the 3-month INT. However, the 3-month INT induced higher increase in salivary HMW-adiponectin concentration, and more favorable changes in BMI, CRF and muscle strength in comparison to the traditional PE classes. Note that changes related to BMI must be interpreted with caution because they could also be affected by growth. Finally, the improvement in CRF induced by the INT was related to the increased post-salivary HMW-adiponectin concentration in these children, explaining 34% of its variance.

In line with the present results that show more favorable BMI in children with higher baseline salivary HMW-adiponectin compared to children with lower baseline salivary HMW-adiponectin, lower plasma adiponectin levels were observed in women with obesity compared to non-obese women (16, 34). Additionally, a previous study in adults has reported that higher plasma adiponectin levels were related to more favorable body composition (15). Studies in children have also shown significantly lower adiponectin concentration in children with overweight and obesity, as compared to non-obese children (35). Considering that the results obtained at the present study are in line with the previous findings, we will accept the first hypothesis and conclude that school-aged children with higher salivary HMW-adiponectin have lower body mass and more favorable BMI than children with lower salivary HMW-adiponectin.

To the best of our knowledge, no previous studies have compared CRF in children depending on their salivary HMW-adiponectin concentration. However, supporting our findings, increased adiponectin concentration was observed in endurance athletes, during the race and at the recovery period (14). The energetic demands of a race require high CRF, which refers to the ability of the cardiorespiratory system to supply oxygen (36), mainly relying on aerobic metabolic pathway for energy production such as oxidative phosphorylation (32). Therefore, more efficient oxidative phosphorylation would result in higher CRF (32). In addition, oxidative phosphorylation has been positively associated with adiponectin concentration (37). In line with previous findings, we assume that the slightly higher CRF in children with higher baseline salivary HMW-adiponectin concentration may have been potentially mediated by the ability for more efficient oxidative phosphorylation (32, 37). However, further experimental studies are necessary to clarify the exact mechanisms and examine the role of oxidative phosphorylation in adiponectin secretion.

We observed only modest changes during the 3-month period in both groups. However, the 3-month INT induced higher increase in salivary HMW-adiponectin as compared to the traditional PE classes, which aligns with the second hypothesis of the present study. To the best of our knowledge, this is the first study to evaluate the effects of an INT program on salivary HMW-adiponectin concentration. INT is a physical exercise program with moderate intensity that improved health and fundamental motor skills in female badminton players (23). Generally, acute and chronic physical exercise with intensities ranging from moderate to vigorous have been shown to induce an increase in adiponectin concentration (12, 18–21). However, some studies reported no significant change in adiponectin levels after physical exercise (14, 22). It was suggested that the different outcomes in the scientific literature may be explained by differences in physical exercise intensity (38). A previous study showed that exercise induced changes in adiponectin concentration in an intensity-dependent manner, with more favorable effects observed after exercise with higher intensity (38). In this line, we believe that the replacement of the traditional warm-up activities during the PE classes in the INT group with the INT program, contributed to an increase from light to moderate intensity, therefore leading to a higher increase in salivary HMW-adiponectin concentration, as compared to the traditional PE classes. Furthermore, a previous study comparing the effects of exercise interventions with different intensities on adiponectin concentration in older adult population has reported that physical exercise-induced changes after the intervention with higher intensity were evident even after 6 months of detraining period (38). This evidence may raise the need for developing further studies that will investigate whether INT-induced increase in HMW-adiponectin concentration in school-aged children would persist after the completion of the INT program. This may be especially relevant, mainly because adiponectin is a cardio-metabolic health protector (12, 13), and the 3-month INT applied in the present study is a cost-effective strategy that can be easily introduced at schools as a strategy for cardio-metabolic health protection and disease prevention.

In addition, the results of the present study indicate that the 3-month INT induced higher improvements in BMI, CRF and muscle strength as compared to the traditional PE classes, which aligns with the third hypothesis of the present study. Even though the higher decrease in body mass and BMI observed in the INT group may result from increased energy expenditure due to higher exercise intensity leading to enhanced lipolysis (39), increased fat oxidation, improved insulin sensitivity and increased glucose uptake by the skeletal muscles (40), we should also consider that these changes may be affected by growth and growth related alterations. On the other hand, the slight improvements in terms of CRF are highly-dependent on the exercise intensity (24). The increased oxygen intake and oxygen delivery induced by exercise intervention with higher intensity may lead to more effective oxygen utilization rate and improved CRF because of the greater muscle capillarization and mitochondrial density (41). Furthermore, strength-related exercises that formed part of the INT program have contributed to slightly higher improvement in muscle strength in the INT group, as compared to the control group which had the traditional PE classes. In this line, a previous study employing a 2-month INT in school-aged children has also reported an increase in upper body muscle strength (24).

With regard to the additional goal of the present study, none of the changes observed in the control group after the 3-month period was related to the post-salivary HMW-adiponectin concentration. Conversely, the slightly higher improvement in CRF induced by the 3-month INT was related to the increased post-salivary HMW-adiponectin concentration in the children from the INT group. Moreover, the improvement in CRF induced by the INT explained 34% of the variance of post-salivary HMW-adiponectin concentration. To the best of our knowledge, there are no studies that explored the direct association between HMW-adiponectin and CRF. However, previous evidence reported that higher adiponectin levels are related to increased maximal oxygen consumption (VO_2_ max) in patients with chronic spinal cord injury (15). VO_2_ max is the total amount of oxygen consumption attainable during physical exertion (42). It is a reliable index measuring the limits of the cardiorespiratory system ability for oxygen transport, and a relevant indicator of CRF (36). Thus, an improvement in CRF is mediated and highly-dependent on the increase in VO_2_ max (36). Based on the previous evidence, we assume that the association between the change in CRF and the increased post-salivary HMW-adiponectin concentration in the children from the INT group, may have been potentially mediated by VO_2_ max (15, 36, 42). We suggest that the slightly higher improvement in CRF in the INT group as compared to the control group, may have been followed by a sufficient increase in VO_2_ max that was enough to induce changes in salivary HMW-adiponectin concentration (15, 36). On the other hand, the intensity of the traditional PE classes in the control group was probably insufficient to induce higher changes in CRF and VO_2_ max that are necessary to impact HMW-adiponectin concentration (24, 38). However, further studies are needed to evaluate the VO_2_ max capacity in laboratory settings and investigate its potential mediatory role in the association between CRF and HMW-adiponectin concentration.

## 5 Conclusion

Children with higher baseline salivary HMW-adiponectin have more favorable BMI and slightly higher CRF than children with lower baseline salivary HMW-adiponectin. Furthermore, big changes after the 3-month period were not observed neither with the traditional PE classes, nor with the INT. However, it might be worthy to note that the INT seems to induce slightly higher increase in salivary HMW-adiponectin, and more favorable changes in BMI, CRF and muscle strength as compared to the traditional PE classes. Finally, the INT-induced improvement in CRF appears to be related to the increased post-salivary HMW-adiponectin concentration after the intervention.

### 5.1 Practical applications

Present findings may serve as a starting point for future research, raising the need for designing studies that may further explore the utility of an INT intervention applied in schools as a strategy to promote health and potentially evoke cardio-protective effects in school-aged children.

These findings may also encourage some modifications in the PE curricula such as the incorporation of an INT in schools with the aim to maintain and potentially enhance the cardio-metabolic health of the children. PE teachers and coaches might also benefit from this study when tailoring exercise programs and training interventions that should provide the optimal conditions for cardio-metabolic health improvement.

### 5.2 Limitations and future research

Nevertheless, the present findings must be interpreted under the consideration of the potential study limitations.

Even though participants complied with the recommendations for physical activity at baseline representing a homogenous sample, there is heterogeneity in terms of type of sport practiced as extracurricular activity (e.g., biking, basketball, swimming etc.). Thus, we must consider this as a potential limitation of the present study. Further interventional studies should consider this and compare the potential effects induced by different sports and extracurricular activities on HMW-adiponectin concentration. These studies may offer new insights into the physiological responses induced by different types of exercise and contribute to the optimization of training interventions that may potentially increase HMW-adiponectin concentration.

Besides the fact that CRF evaluation was performed by experienced researchers in the present study, the timing was manual, thus it should be considered as a potential study limitation as well. Future studies should consider including automatic timing in CRF evaluation.

Another limitation could be the nature of the present study which does not allow us to elucidate further the molecular mechanisms underlying the relation between CRF and post-salivary HMW adiponectin concentration. However, obtained results are raising the need for designing future studies that will investigate the VO_2_ max capacity in laboratory settings and examine its role in mediating the association between CRF and salivary HMW adiponectin concentration. We also believe that further animal studies may provide even more valuable insights into the exact physiological pathways that are involved in regulation of the HMW-adiponectin secretion.

Finally, we also believe that larger comparative studies in the future should focus on sex-specific comparisons in HMW-adiponectin concentration. This kind of studies may provide insights into the biological mechanisms that underlie HMW-adiponectin regulation, and potentially contribute to more effective and personalized therapeutic interventions.

## Data availability statement

The original contributions presented in the study are included in the article/Supplementary material, further inquiries can be directed to the corresponding author.

## Ethics statement

The studies involving humans were approved by Institutional Review Board of Dr. Josep Trueta Hospital, Girona, Spain. The studies were conducted in accordance with the local legislation and institutional requirements. Written informed consent for participation in this study was provided by the participants’ legal guardians/next of kin.

## Author contributions

FV: Conceptualization, Data curation, Formal analysis, Investigation, Methodology, Software, Writing – original draft. RF-L: Conceptualization, Data curation, Formal analysis, Funding acquisition, Investigation, Methodology, Project administration, Software, Supervision, Writing – review & editing. GC-B: Formal analysis, Methodology, Software, Writing – review & editing. JC-G: Data curation, Formal analysis, Methodology, Writing – review & editing. AL-B: Formal analysis, Investigation, Methodology, Supervision, Writing – review & editing. AP-P: Conceptualization, Data curation, Formal analysis, Funding acquisition, Investigation, Methodology, Project administration, Software, Supervision, Writing – review & editing.
