# Supplementary material for: Integrated neuromuscular training intervention applied in schools induces a higher increase in salivary high molecular weight adiponectin and a more favorable body mass index, cardiorespiratory fitness and muscle strength in children as compared to the traditional physical education classes
Source: Front Public Health. 2024 May 2;12:1337958. doi: 10.3389/fpubh.2024.1337958 (PMC11096568; doi:10.3389/fpubh.2024.1337958)
Supplement: Supplementary file 1 [file Table_1.DOCX]

Supplementary Table S1. Baseline characteristics of the studied population and comparison between the control and the INT group

| Baseline characteristics | control group  (N=45) | INT group  (N=45) | p-value |
| --- | --- | --- | --- |
| HMW-adiponectin (ng/ml) | 7.17 (3.56 - 9.42) | 5.66 (2.34 - 8.32) | 0.214 |
| age (years) | 7.39±0.37 | 7.48±0.33 | 0.230 |
| sex (m/f) | 24/21 | 19/26 | 0.240 |
| body mass (kg) | 25.60±3.81 | 25.72±4.70 | 0.904 |
| body mass SDS | -0.28 (-0.79 - 0.13) | -0.44 (-0.81 - 0.21) | 0.860 |
| height (cm) | 126.37±6.41 | 126.36±5.84 | 0.994 |
| height SDS | -0.09 (-0.54 - 0.88) | -0.05 (-0.57 - 0.85) | 0.909 |
| BMI (kg/m2) | 16.14±1.68 | 16.23±2.28 | 0.842 |
| BMI SDS | -0.42 (-0.75 - 0.10) | -0.31 (-0.77 - 0.03) | 0.746 |
| CRF (min)* | 5.33±0.49 | 5.29±0.62 | 0.788 |
| muscle strength (kg) | 10.46±2.25 | 9.76±2.19 | 0.163 |
| Data for Gaussian variables is presented as mean ± standard deviation. Data for non-Gaussian variables is presented as median and interquartile range. The p-value for Gaussian variables is from t-test. The p-value for non-Gaussian variables is from Mann-Whitney U test. The p-value for categorical variables is from Chi-squared test. Significance level is set at 0.05. BMI: body mass index; CRF: cardiorespiratory fitness; HMW: high molecular weight; INT: integrated neuromuscular training; SDS: standard deviation score; *: variable with an opposite metric orientation. | | | |
